# Standardized studies of the oral microbiome: From technology‐driven to hypothesis‐driven

**DOI:** 10.1002/imt2.19

**Published:** 2022-04-11

**Authors:** Chuqi Gao, Xuantao Li, Xiaole Zhao, Peiyue Yang, Xiao Wang, Xiaoli Chen, Ning Chen, Feng Chen

**Affiliations:** ^1^ Central Laboratory Peking University Hospital of Stomatology Beijing China; ^2^ Department of Gastroenterology Peking University People's Hospital Beijing China

**Keywords:** hypothesis‐driven, metagenomics, oral microbiome, pathogenesis, technology‐driven

## Abstract

The microbiome is in a symbiotic relationship with the host. Among the microbial consortia in the human body, that in the oral cavity is complex. Instead of repeatedly confirming biomarkers of oral and systemic diseases, recent studies have focused on a unified clinical diagnostic standard in microbiology that reduces the heterogeneity caused by individual discrepancies. Research has also been conducted on other topics of greater clinical importance, including bacterial pathogenesis, and the effects of drugs and treatments. In this review, we divide existing research into technology‐driven and hypothesis‐driven, according to whether there is a clear research hypothesis. This classification allows the demonstration of shifts in the direction of oral microbiology research. Based on the shifts, we suggested that establishing clear hypotheses may be the solution to major research challenges.

## Highlights

- Genomics technologies have undoubtedly boosted the process of oral microbiology research which broadens people's understanding of diseases.
- Studies driven by genomics technologies tend to obtain a microbiological portrait of a specific population and then describe it.
- However, the recent studies share some goals even more concrete and definite, which symbolizes a critical turning point of global trend naturally from technology‐driven to hypothesis‐driven.
- Hypothesis‐driven studies have currently focused on the validation of pathogenic mechanisms and the effectiveness evaluation of therapeutics.

## Graphical abstract

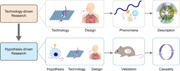

## INTRODUCTION

The microbiome can be regarded as a second human genome, with which we are in a symbiotic relationship [1, 2]. We have just begun to reveal the nature and strength of this relationship, as well as its influence on physiology and pathology. Among the microbial consortia in the human body, that in the oral cavity is singularly complicated, with the second greatest diversity [3]. The oral cavity connects the outside with the digestive and respiratory tracts, and the microorganisms therein protect against harmful external factors. Microbiota dysbiosis is associated with oral diseases, such as periodontitis [4], peri‐implantitis [5], oral mucosal diseases [6], and dental caries [7], as well as systemic diseases, including gastrointestinal, endocrine, immune, and neurological diseases [8–11].

Due to the lack of specific driving factors (e.g., smoking, alcohol consumption, and human papillomavirus) for some diseases, scholarly attention is drawn to oral microorganisms as potential risk factors [12]. Furthermore, understanding the oral microbiome is essential for explaining the role of other risk factors in disease development.

The advent of next‐generation sequencing (NGS) has pushed the number of publications in the sequencing‐based oral microbiome to an unprecedented level, a breakthrough in human oral microbiology. To date, there has been a critical shift in research direction.

## TECHNOLOGY‐DRIVEN RESEARCH PROGRESS

**Figure 1:**
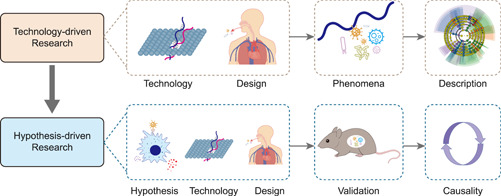
Technology‐driven studies refer to descriptive ones. That is, the study utilizes next‐generation sequencing technology to obtain a microbiological portrait of a specific population, and then describes it. Designing experiments to validate and thereby derive causality is called hypothesis‐driven research

Describing the microbiology of a disease helps to infer its pathogenesis, namely extrapolating causality from phenomena (Figure 1). Technology‐driven research focuses on expanding databases, new advances and methods in sequencing technologies, as well as identifying new target populations and factors correlated with diseases.

### Expanded database

The Human Oral Microbiome Database (HOMD) is based on 16S ribosomal RNA (rRNA) gene reference sequences and is a valuable resource. The expanded Human Oral Microbiome Database (eHOMD) covers numerous microbial species inhabiting the mouth and nose [13]. However, annotation or classification is hampered by the high conservation of 16S sequences among closely related species. For this reason, an intermediate taxonomic “superspecies level” was added between genus and species, which consisted of indistinguishable species sharing closely related sequences, reducing the error rate of short‐ and long‐read 16S rRNA [14].

However, the HOMD is derived from microbial culture data. Because of culture conditions and interactions of the microbiome with other factors, 20%–60% of oral microorganisms in the HOMD are unculturable [15]. Sequencing the metagenomes of bacteria isolated from the oral cavity enables the recognition of some unculturable oral microbes [16]. Using metagenomic shotgun data for 3346 oral metagenomics samples together with 808 published samples, 56,213 metagenome‐assembled genomes were obtained. Over 64% of the 3589 species‐level genome bins contained no publicly available genomes [17]. This study makes an important contribution to the replenishment of the database.

Using tongue and plaque samples, 790 nonredundant genomes were reconstructed, 43 of which belonged to *TM7*, forming six monophyletic clades [18]. The diversity of *TM7s* and their link to oral mucosal infectious diseases have been reported [19]. Moreover, 42 new sites (plaque vs. tongue) specific to *TM7* were identified in 47 human samples, including the *Saccharibacteria clades G3* and *G6* [20]. The whole‐genome sequence of *C17T* was isolated from a child's oral cavity by Qi et al. [21] and an almost‐complete genome of a *Tannerella* sp. *BU045* was detected by DNA amplification and sequencing from a single bacterial cell [22]. In a long‐read metagenomic study based on PromethION, 10 jumbo oral phages and prophages in human saliva were selected, the same as plasmid‐like components [23].

In addition to the discovery of new taxa, metagenome assembly can assemble the genomes of uncultured bacteria. Large‐scale metagenomic data from massive samples also allow the assembly of strains from important oral taxa, such as *Porphyromonas* and *Neisseria* [17]. In the assembled overlap, 50% of genes were singletons (unique to a single metagenomic sample). This clarified the unexplained heterogeneity in microbiome‐derived human phenotypes [16].

**Figure 2:**
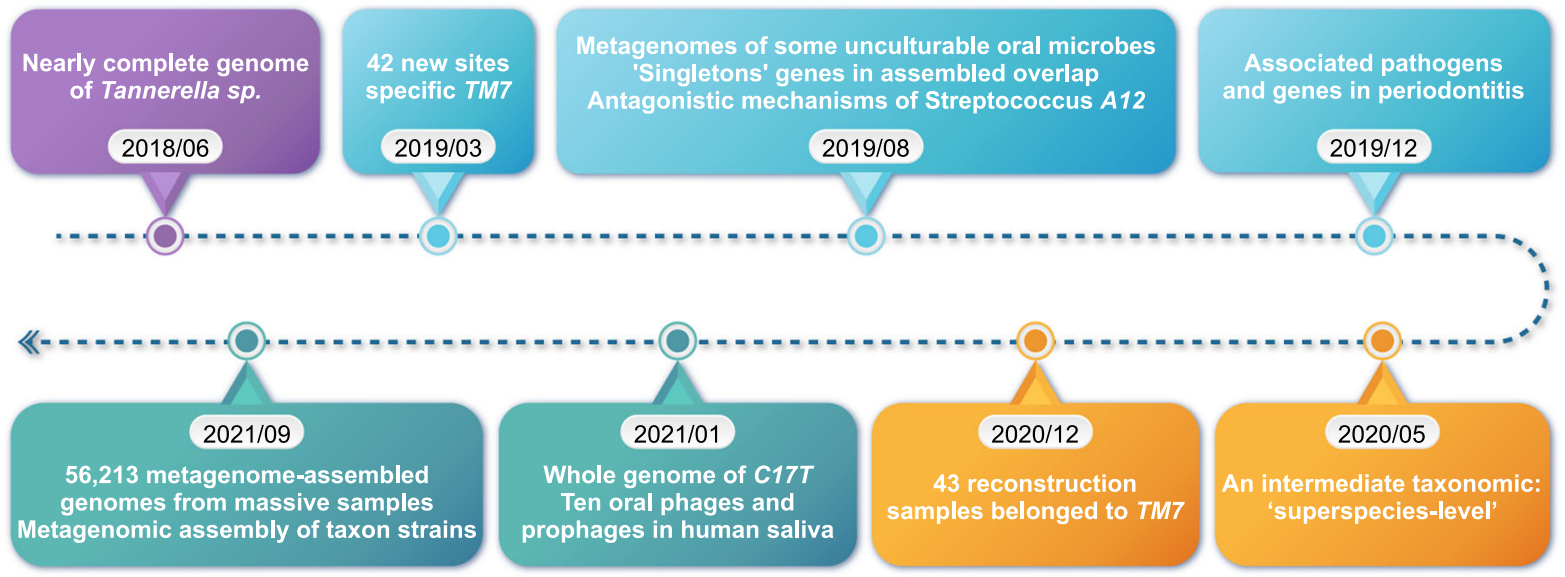
Human Oral Microbiome Database is constantly expanded and updated. Here is the database development from June 2018 to September 2021

There are also previously isolated bacteria whose function is unclear, such as *Streptococcus* sp. *A12*. The molecular mechanisms by which this microbe resists antagonistic factors were identified by functional genomics, facilitating the development of biomarkers and therapeutics [24]. There is an association between the phylum *Saccharibacteria* and oral mucosal infectious diseases [19]. Interestingly, many oral communities contain homologs to gut bacteria encoding enzymes relating up to 41 human targeted drugs [17]. The HOMD is constantly updated with new discoveries (Figure 2).

### New methods and technologies

Optimization and improvement of research methods are critical in this field. Yano et al. compared the human oral microbiome using four sample‐collection methods, which had a marked influence on the oral microbiome [25]. To overcome this variability, osmotic lysis followed by propidium monoazide treatment increases the yield of microbial DNA from human oral samples. It might be extended to other sample types [26].

A low‐cost sequencing method with higher resolution than 16S rRNA gene sequencing has been developed. The resolution of amplicon‐based microbiome identification was increased by merging high‐diversity marker gene sequencing (ribosomal 16–23S intergenic spacer region) and the DADA2 probabilistic error modeling based denoising algorithm [27].

Advances have been made in statistical methods for oral microbial genomics. As a mature method [28], co‐occurrence network analysis has been used in oral microbiology to evaluate the associations between the oral microbiome and other habitats, as well as the relationship between the oral microbiome and metabolites [20, 29]. Similarly, genome‐scale modeling has been used to identify microbiomes in a variety of tissues [30].

Machine learning algorithms can be used to recognize oral microbiome species from buccal and supragingival sites, representing those in subgingival plaque. This microbiota holds promise as a marker for the early diagnosis of periodontal disease [31].

Several novel techniques have been applied in oral microbiology. Based on 16S rRNA gene sequencing, the Human Oral Microbe Identification Microarray (HOMIM) can simultaneously detect about 300 of the most common oral bacterial species, including several nonculturable taxa [32]. HOMI NGS is a more comprehensive semiquantitative technique than HOMIM, allowing better characterization of oral microorganisms [33].

However, 16S rRNA gene sequencing is limited to 16S rRNA fragments, whereas identification of microorganisms should be based on the entire genome. Multiomics techniques (e.g., gene, transcription, proteomics) have been applied to oral microbial genomes [34]. Combining public genomes with Human Microbiome Project metagenomes, differences in the distribution of bacterial flora in the dorsal tongue, buccal mucosa, and supragingival plaque have been found [35]. Moreover, proteomics and metabolomics indicated systematic differences between the plaque and calculus microbiome, which was associated with biofilm physiology [36]. Multiomics will increasingly be used in microbiology studies.

The combination of traditional in vitro animal models and genomics has much potential. Adult *Macaca mulatta* were used to explore the interaction between oral microorganisms and gene expression profiles in autophagy, hypoxia, and apoptosis [37]. Fecal transplants have been used in studies of gut microorganisms [38], hinting at an innovative idea of transplanting oral bacteria from patients into germ‐free mice. Replication or transplantation of a healthy oral microbiota into patients has therapeutic potential for oral and systemic diseases. However, to date, no microbiological evaluation or clinical assessment has been performed.

Thus, new research methods and technologies facilitate the studies of microorganisms. The combination of omics with other technologies, or the application of advanced technologies in other fields, may trigger a new phase of microbiological research.

### New research populations

**Figure 3:**
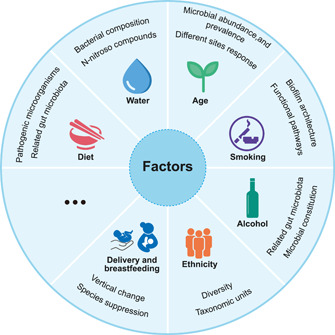
The investigation of the factors influencing oral microbiology continues, including age, smoking, alcohol, ethnicity, diet, water, delivery, breastfeeding, and so on. The studies included are all conducted using next‐generation sequencing technologies. It is evident that many studies are no longer limited to the observation of diversity and species distribution, but are gradually moving toward structural, mechanistic, or longitudinal perspectives, which will provide a clearer picture of the clinical significance of these factors.

The baseline levels of the oral microbiome in populations with different epidemiological characteristics have been reported, expanding our understanding of oral microorganisms (Figure 3).

Microbiome revealed a precise and perceptible association with age, including the number, abundance, and prevalence [39]. Microbial communities within different sites show a bell‐shaped trend in response to aging [40]. Human groups in different regions vary greatly in the degree of diversity among and within individuals [41]. The 28 species‐level operational taxonomic units vary considerably among different ethnic groups in Canada [42].

Smokers and e‐cigarette users were both rich in pathogens in their oral cavities, and the most significant effect was the changing of biofilm architecture [43]. Smoking also may lead to shifts in functional pathways that have an impact on smoking‐related diseases [44]. The level and type of drinking alcohol were associated with the overall microbial constitution and individual taxon abundance [45]. The Shannon diversity was lower in the oral microbiome than in the gut [46]. Drinking tap water might play a significant part in shaping oral microbiota, and the composition of tap water had a connection with the major modifications in the abundance of several bacterial genera [47]. Drinking high nitrate water increases oral nitrate‐reducing bacteria, which may lead to an increase in N‐nitroso compounds [48].

Differences in the composition of the salivary microbiota of vegetarians and omnivores exist at all taxonomic levels below the phylum level, including species associated with periodontal disease [49]. Oral and gut microbiota were correlated with specific dietary components, such as vegetable and sweets intake [50]. A relationship was confirmed between the mode of delivery and primitive bacterial content in the oral microbiome. During the first 2 years of age, shorter breastfeeding time and whether antibiotic‐treated were related to a distinct bacterial composition at a later age [51]. Oral microbiota differs between breast‐fed and formula‐fed infants at 3 months of age [52].

Human oral microbiological characteristics are influenced by multiple factors, but the underlying mechanism is unknown. For example, the salivary microbiome of obese subjects is distinct from that of nonobese subjects. Džunková et al. performed a longitudinal analysis of saliva samples from obese adults who underwent bariatric surgery. Various individual‐specific factors other than weight influenced the salivary microbiome distribution. It is reasonable for further studies to focus on the correlation between altered taste preferences and latent oral health deficit [53]. There are some other factors that affect the microbiome lack of causality. Various habits associated with modern lifestyles can reduce the diversity of the human oral microbiome, such as high‐sugar diets, alcohol consumption, smoking, and other factors [54]. For another instance, the pathological processes of mother‐to‐child microbiome transmission remain unclear [51, 55, 56]. In addition, the oral microbiota of smokers and e‐cigarette users contain pathogens, but with different bacterial compositions [43]. The studies reflect the unexplained heterogeneity in human microbiomes [16]. It is necessary to explain how such heterogeneities arise, which can be overcome by expanding research populations by including hitherto ignored groups, such as adolescents and babies [47].

### New descriptions of disease

The association of some diseases with oral microorganisms has been updated, such as Alzheimer's disease (AD), Parkinson's disease (PD), and gestational diabetes (GDM).

There is a strong correlation between AD and the oral microbiota, and the impaired oral motor skills and inability to perform oral care as a result of cognitive impairment in patients with AD increases the risk of dental caries and periodontal disease [11]. Holmer et al. reported that the subgingival microbiota showed typical periodontal disease features in individuals with cognitive impairment or AD [57]. Fleury et al. sampled the microbiota of saliva and subgingival plaque and, using 16S rRNA gene amplicon sequencing, found that the oral microbiome changed in early and midterm PD patients, which might be related to local inflammation in the oral cavity [58]. Using 16S rDNA sequencing, Xu et al. discovered a link between GDM status in the third gestational period and oral microorganisms during pregnancy. The changes in the intestinal and oral microbial community might be used as noninvasive biomarkers for monitoring GDM in pregnancy [43, 59]. A common limitation of these studies is that they focus on correlation rather than causality. However, although the causality between these diseases and oral microbiota has not been confirmed, such correlations could help diagnose and prevent diseases.

There are limitations in studies of some other diseases, such as adverse pregnancy outcomes (APO). The clinical evidence of immunological activity in APO is dependent on a cross‐sectional case‐control studies. However, the gestation period includes proinflammatory and anti‐inflammatory phases, both of which are affected by fluctuations of female sex hormones [60]. This leaves such studies lacking in potential for future mechanistic exploration.

On the other hand, genomics technology has provided a clearer picture of human oral microbial genes, increasing our understanding of the disease. Human‐associated microbe pangenomes have been expanded by the thousands of microbial genomes of yet‐to‐be‐named species identified [61]. The first global snapshot of the healthy oral microbiome and its resistome was obtained from the data of 20 subjects by whole‐genome sequencing and microarray analysis [62]. For the first time, a catalog of microbiome genes from children with caries was constructed using samples from 25 3–5‐year‐old preschool children suffering from severe early childhood caries and 19 age‐matched healthy controls [63]. Also, 16S rRNA sequencing provided insight into the apical periodontitis and Down syndrome microbiome [64, 65]. With known findings previously into the biogeographic variation, an anterior‐to‐posterior gradient of subgingival community composition was determined [66]. However, most of these studies were small, and larger‐scale confirmatory studies are needed.

### New diagnostic tools and models

Interindividual differences hamper generalization of the findings of microbiome studies. This means that it is not reliable to predict diseases only by the abnormal distribution of individual microorganisms unified standard is needed.

However, genetic diversity has not been completely quantified. Tierney et al. conducted a cross‐study meta‐analysis on metagenomes from two human‐body niches, with 3655 samples from 13 studies, and identified 23,961,508 oral and 22,254,436 gut nonredundant genes among a total of 45,666,334. The data were used to create a resource (https://microbial-genes.bio/) [16, 55].

Oral microbiological diagnostic tools and predictive models have been proposed based on large‐scale studies. The large sample size and several statistical methods reduce the interference of individual discrepancies in the results of those studies, namely the standardization process. With the standardization, the reliability of disease diagnosis tools and prediction models could be relatively trusted. The oral microbiota composition differed significantly between primary Sjögren syndrome and systemic lupus erythematosus patients, suggesting that it can be used to distinguish the two [67]. Xu et al. conducted a 1‐year longitudinal observation by 16S rDNA sequencing of the occurrence and development of caries in 144 3‐year‐old children, including 10 with caries and 19 healthy children as controls. The accuracy of their prediction model reached 93.1% [68]. Considering the high prevalence of dental caries, microbiological diagnostic tools are important, which requires a unified standardization of individual differences.

## HYPOTHESIS‐DRIVEN RESEARCH PROGRESS

It is essential to formulate a potentially plausible hypothesis based on theoretical and scientific experience, which can be verified experimentally or clinically (Figure 1). The isolation of new bacteria, the recognition of new research populations, and links between diseases and the oral microbiome lay the foundation for hypothesis‐driven research.

By conducting confirmatory experiments, hypothesis‐driven research can lead to conclusive causality about the microbiological or immunological mechanisms of disease, the effectiveness of treatments, and so on.

### Oral microbiome in disease pathogenesis

The complex metabolic and functional interactions within dental biofilms and between them and the host are involved in health‐maintaining mechanisms [69]. Wang et al. longitudinally tracked the reassembly of human oral biofilms after disturbance. The data revealed the resilience and long‐term stability of the oral microbial community and the critical time points and stages of the dramatic transformations in, and structural recovery of, the microflora [70].

There is an unambiguous relationship between oral microbial metabolism and oral diseases, such as dental caries [7], periodontitis [5], and oral mucosal diseases [71]. The theory of oral diseases has changed from a single pathogen to microecological dysbiosis. Dysbiosis is a result of the interactions of bacteria, fungi, and viruses in the community. For example, in severe early childhood caries etiopathogenesis, the activity of glucosyltransferases in plaque was significantly elevated by *Candida albicans* [72].

Genomics technology has been used in immunological research on pathogenic mechanisms, resulting in the discovery of putative pathogens and novel genes not previously linked to periodontitis [73]. A longitudinal study provided evidence that genes involved in carbohydrate‐related metabolism, such as methane metabolism, and energy‐metabolism‐related parameters were enriched in late‐stage oral squamous cell carcinoma, whereas those responsible for amino acid metabolism were significantly associated with the healthy controls [74]. Taste‐preference‐associated genes were found to have interrelationships with sucrose ingestion as well as allelic variation, using hierarchical cluster analysis of salivary microbiota groups [75]. Recent bioinformatics research has focused on gene clusters generating micromolecules, suggesting relationships between the microbiological community and signal molecules [76]. For instance, catechol siderophore synthesis gene clusters were detected in both *Rothia mucilaginosa* and *Rothia dentocarios* cultured in the presence of glycerol, which represented rich *R. mucilaginosa* in the saliva of children in health conditions [77]. Mohan et al. explored metatranscriptome data sets to assess the RNA regulatory mechanisms and metabolic shifts [78]. These gene‐level studies have provided insights on previously known pathways as they relate to oral cavities, with the aim of building a more comprehensive model of the oral microbial landscape.

There is a consensus that periodontal diseases are related to the oral microbiome. As the result of the host immune disorders, inflammation provides a suitable nutritional environment for pathogens and further promotes the persistence of dysbiosis. Thus, periodontitis is driven by a self‐sustainable cycle in which inflammation and dysbiosis positively strengthen each other. As an inflammatory response, periodontal disease is a typical dynamic process indicating the interaction between oral microorganisms and the host [79], and follow‐up studies should pay more attention to this dynamic nature to better understand the disease pathogenesis.

Oral dysbiosis is hypothesized to result in diseases of the gastrointestinal, endocrine, immune, and nervous systems [8–11]. Syndromic chronic periodontitis is a form of chronic periodontal damage that is considered a symptom of systemic disease as a result of defects in key genes that affect periodontal structures or immunity [80]. Dysbiosis can also cause periodontitis, likely resulting in bacteremia and the exacerbation of systemic diseases [81]. In conclusion, there is a two‐way interaction between oral microbiota and systemic diseases.

Systemic diseases are now in a reinforced causality with oral microbiome on the perspective of bacterial transplantation. Medini et al. evaluated cellular and soluble markers of inflammation and immune malfunction and found that the bacterial and fungal oral microbiome might be involved in chronic systemic immune activation in HIV‐infected patients [10]. Moentadj et al. researched the oral microbial community in a prospective cohort of rheumatoid arthritis (RA) patients, first‐degree relatives, and healthy controls (HC). Mild chronic arthritis in mice was induced by the streptococcal cell wall from RA and HC‐associated *S. parasalivarius* strains [82].

The oral microbiome may drive the development and exacerbation of systemic diseases via its action on the gut. For example, the oral microbiome is overrepresented in the lower intestinal tract in liver cirrhosis, potentially contributing to disease development and severity [83]. Intestinal inflammation is a causative factor for systemic diseases, including arthritis, psoriasis, and uveitis [84]. However, whether this is a result of oral inflammation or a direct effect of oral bacteria is unclear [85].

Periodontal inflammation reportedly contributes to gut inflammation in vivo. Human TH17 cell defects were correlated with reduced periodontal inflammation and bone loss [86]. Kitamoto et al. used mouse periodontitis and enteritis models to show that periodontitis produces oral phobia‐responsive Th17 cells which migrate to the gut and trigger colitis [8]. Also, *Klebsiella* spp. are strong inducers of T helper 1 cells in mice [87].

**Figure 4:**
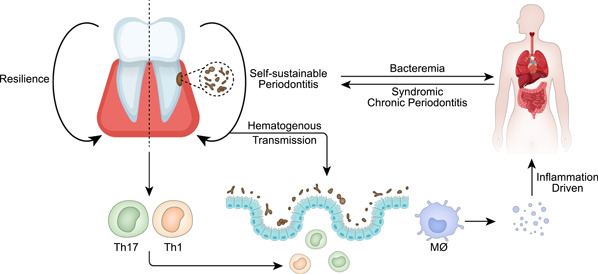
Potential mechanisms for the association of oral diseases with systemic diseases. A healthy oral cavity is maintained through resilience. Oral inflammation is self‐sustainable and bidirectionally driven to systemic disease. The mechanism may be that periodontal bacteria can enter the intestinal tract through the hematological circulation to trigger inflammation, or it can induce Th17, Th1, and other cells in the oral cavity to make their way to the intestinal tract. The inflammatory factors produced by inflammation lend themselves to an immune response to induce systemic disease

The oral cavity acts as a reservoir for gut bacteria [88], and oral‐intestinal microbial transfer is accepted. A multistage model of bacterial intestinal transport from the oral cavity emphasized the importance of the oral–intestinal axis microbial and immune compartments [86]. Hematogenous transmission is likely preferred according to Abed et al. in a study of oral fusobacteria translocating to colon tumors [89]. Ingestion or transplantation into the gut of *Klebsiella* and *Enterobacter* caused inflammasome activation in colonic mononuclear phagocytes and inflammation [8] (Figure 4).

Metagenome sequencing has shown that the oral microbiome is significantly associated with cancer, suggesting novel prophylactic approaches. Possible mechanisms include upregulating putative virulence factors related to membrane biosynthesis, flagellum synthesis and assembly, iron transport, chemotaxis, hemolysin, and adhesins [90]. Other such studies have focused on oral [91–93], colorectal [89, 94, 95], pancreatic [96, 97], lung [98–100], and esophageal [101–103] cancer.

Multiple biological and environmental factors influence disease susceptibility, development, and severity, but longitudinal studies on these effects are scarce. Freire et al. analyzed the supragingival plaque microbiomes of dizygotic and monozygotic twins in a longitudinal study and found that oral microbiome variation was mainly caused by the environment [104]. Also, infants born by Cesarean section initially had abnormal levels of bacteria compared to those delivered vaginally, but this recovered with age. This finding revealed that oral microbiota development is an ecological succession [16].

Surana et al. delineated a bioinformatically straightforward approach to triangulate microbiota members likely to influence disease pathogenesis, enabling the sequencing methods to meet Koch's postulates [105]. Sanna et al. and Zhuang et al. employed bidirectional Mendelian randomization analyses to assess causality [106, 107]. These methods could be applied to demonstrate the causality of links between the oral microbiome and disease in the future.

### Oral microbiome in disease therapy

Inflammatory bowel disease involves the oral intestinal microbial transfer pathway. Drug therapy is aimed at rectifying dysbiosis of inflammatory bowel disease. According to Simon‐Soro et al., drug effects, such as the intervention of thonzonium bromide in the oral cavity and gut, were impervious to each other. However, different oral flora could colonize the intestinal tract [9]. One possible explanation is that the drug intervention on the oral microbial community is in the original site of action, which affects other communities in the whole human body. A healthy oral microbiome could have therapeutic potential for gastrointestinal disease. In another study, the salivary microbiota colonized the gut, including *Klebsiella* strains resistant to several antibiotics. The study identified members of the healthy intestinal microbiome that resisted oral bacterial colonization, promoting the development of drugs active against resistant bacteria and for chronic inflammation [87].

Oral microorganisms can guide cancer treatment. A loss of alpha diversity was found in the stool and oral samples of acute myeloid leukemia (AML) patients receiving carbapenems [108]. In a prospective cohort analysis of 90 patients with AML, no association of *Stenotrophomonas maltophilia* infection with fecal relative abundance was found. Instead, the oral microbiome was predictive of *S. maltophilia* infection in chemotherapy patients with AML. Researchers also found that cumulative meropenem exposure was linked to an increased infection risk [109]. These conclusions will assist in balancing the efficacy and indirect damage of broad‐spectrum antibiotics in cancer treatment.

Oral mucositis (OM) is one of the most common complications of chemotherapy. In cancer therapy, a dynamic oral microbial community can trigger cancer therapy‐induced oral mucositis. In patients with locoregional squamous cell carcinoma of the head and neck, there was an association between the abundance of microbial genera and the occurrence of severe OM during treatment [110]. Hong et al. performed metagenomic sequencing of 49 participants undergoing 5‐fluorouracil or doxorubicin‐based chemotherapy and 30 healthy subjects longitudinally during one cycle. The results indicated that chemotherapy‐induced OM and dysbiosis were closely linked, and inflammation‐associated dysbiotic shifts could aggravate the injury to the oral epithelium. Controlling oral bacterial dysbiosis has the potential for improving oral mucositis [6].

In short, technology‐driven research has generated extensive databases, greatly broadening our knowledge of the oral microbiome. And hypothesis‐driven research focuses on scientific problems, transforming microbiological knowledge into useful clinical tools.

## CONCLUSIONS

Oral microbiology research has reached a bottleneck. Instead of repeatedly confirming biomarkers of oral and systemic diseases, recent studies have focused on developing a unified clinical diagnostic standard in microbiology that reduces the effect of interindividual differences. Research has also focused on the mechanisms of bacterial pathogenesis and the effects of treatments. There are two major challenges in oral microbiology research.

One is the demand for hypothesis‐driven rather than technology‐driven research. Technology‐driven studies lack clear hypotheses but rather explore blank areas, resulting in contradictory or clinically nonsignificant conclusions. In contrast, hypothesis‐driven studies are likely to show greater clinical significance because of their concrete purpose and use of the databases created in the past two decades. Therefore, microbiome research should be hypothesis‐driven rather than technology‐driven. This is the first time for this classification method to be proposed. It clearly demonstrates a principal shift in the direction of oral microbiology research.

Another important challenge is the insufficient sample size. NGS has enabled the creation of large databases and has revealed marked interindividual variability. Therefore, standardization is important to deal with the data sets created. Most prior reviews of oral microbiology overlooked standardization, and others covered it only at the conceptual level. We emphasize the importance of standardization and the use of clear hypotheses in future studies.

The influence of oral microorganisms on tissues, organs, and systems may affect the whole body, thereby not only interfering with the judgment of the impact of a single disease but also providing new ideas for drug therapy. Thus, the oral microbiome is of great potential, whether in terms of disease prevention, diagnosis, or treatment. However, there is a lack of attention to the oral microbiome compared to the gut microbiome. Although they are correlated, phenomena linked to gut microbes may not be applicable to oral microbes. Therefore, the purpose of this review is to guide researchers who want to approach the study of the oral microbiome in the most reasoned and effective way, to speed up the process of oral microbial research.

## AUTHOR CONTRIBUTIONS

Chuqi Gao, contributed to the conception, design, drafting, interpretation, and critical revision of the manuscript. Xiaole Zhao, contributed to drafting, interpretation, and revision of the manuscript. Xuantao Li, contributed to analysis, design, drafting, interpretation, and critical revision of the manuscript. Peiyue Yang, contributed to the analysis, drafting, interpretation, and revision of the manuscript. Xiao Wang, contributed to the design, drafting, interpretation, and critical revision of the manuscript. Xiaoli Chen, contributed to the design, drafting, interpretation, and critical revision the manuscript. Feng Chen and Ning Chen, contributed to the conception, design, and critical revision of the manuscript. All authors gave their final approval and agreed to be accountable for all aspects of the work.

## CONFLICTS OF INTEREST

The authors declare no conflicts of interest.

## Data Availability

Data sharing is not applicable to this article as no data sets were generated or analyzed during the current study. Supplementary information (graphical abstract, slides, videos, Chinese translated version, and updated materials) are available online at DOI or http://www.imeta.science/.
